# 受者年龄对异基因造血干细胞移植结局的影响：一项来自中国骨髓移植登记组（CBMTRG）的分析

**DOI:** 10.3760/cma.j.cn121090-20260324-00150

**Published:** 2026-07

**Authors:** 晓利 梁, 竞 刘, 丽 宣, 耀 孙, 佳 陈, 佳 刘, 海霞 付, 聪聪 王, 晟晔 卢, 兰平 许, 筱淇 王, 瑞昊 黄, 德沛 吴, 代红 刘, 启发 刘, 曦 张, 晓军 黄

**Affiliations:** 1 陆军军医大学第二附属医院（新桥医院）血液病医学中心，陆军军医大学第二附属医院（新桥医院）血液生态与智慧细胞科学创新中心，解放军血液病中心、全军临床重点专科、重庆市临床重点专科，重庆 400037 Medical Center of Hematology, Xinqiao Hospital of Army Medical University, Innovation Center for Hematology Ecology and Intelligent Cell Science of Xinqiao Hospital of Army Medical University, PLA Center for Hematology, National Key Clinical Discipline of PLA, Chongqing Key Clinical Discipline, Chongqing 400037, China; 2 北京大学人民医院，北京大学血液病研究所，国家血液系统疾病临床医学研究中心，北京市造血干细胞移植重点实验室，北京 100044 Peking University People's Hospital, Peking University Institute of Hematology, National Clinical Research Center for Hematologic Disease, Beijing Key Laboratory of Hematopoietic Stem Cell Transplantation, Beijing 100044, China; 3 南方医科大学血液病研究所，广州 510515 Institute of Hematology, Southern Medical University, Guangzhou 510515, China; 4 解放军总医院第五医学中心血液科，北京 100853 Department of Hematology, the Fifth Medical Center of PLA General Hospital, Beijing 100853, China; 5 苏州大学附属第一医院血液科，苏州 215006 Department of Hematology, the First Affiliated Hospital of Soochow University, Suzhou 215006, China

**Keywords:** 异基因造血干细胞移植, 年龄, 倾向性评分匹配, 老年患者, 预后, Allogeneic hematopoietic stem cell transplantation, Age, Propensity score matching, Elderly patients, Prognosis

## Abstract

**目的:**

探讨均衡混杂因素后，受者年龄是否独立影响异基因造血干细胞移植（allo-HSCT）预后及其主导的失败模式。

**方法:**

本研究为一项回顾性、多中心队列研究。收集5个移植中心2018年1月1日至2023年12月31日的单次allo-HSCT患者1 619例，分为年轻受者组（≤35岁）与老年受者组（≥50岁）。采用倾向性评分匹配均衡11项协变量后，694例（每组347例）纳入分析。比较两组总生存（OS）率、非复发死亡率（NRM）、累积复发率（CIR）及急慢性移植物抗宿主病（aGVHD/cGVHD）发生率。

**结果:**

中位随访33.4（95％ *CI*：31.2～36.1）个月。老年受者组3年OS率显著低于年轻受者组［（75.5±2.5）％对（83.1±2.2）％，*HR*＝1.59，*P*＝0.007］；3年NRM风险呈升高趋势［（13.3±1.9）％对（8.5±1.6）％，单因素*SHR*＝1.61，*P*＝0.044］，但在多因素校正后未达传统统计学显著性（*aSHR*＝1.59，95％ *CI*：0.99～2.54，*P*＝0.053），效应量稳定提示临床意义。两组CIR、Ⅱ～Ⅳ度aGVHD及cGVHD累积发生率差异均无统计学意义。其中，感染是两组最主要的非复发死亡原因，老年受者组因器官功能障碍及出血死亡的比例有升高趋势。

**结论:**

在倾向性评分匹配后，受者高龄仍与较差OS独立相关；其不良结局主要表现为NRM风险升高，而不表现为复发风险增加。

## Abstract (en)

**Objective:**

To investigate whether recipient age independently affects outcomes after allogeneic hematopoietic stem cell transplantation（allo-HSCT）, including the predominant patterns of treatment failure, after adjustment for confounding factors.

**Methods:**

This retrospective, multicenter cohort study included 1 619 patients who underwent a single allo-HSCT at five transplant centers from January 1 2018, to December 31 2023. Patients were categorized into younger（≤35 years）or older recipient groups（≥50 years）. After propensity score matching to balance 11 covariates, the analysis included 694 patients（347 in each group）. Overall survival（OS）, non-relapse mortality（NRM）, cumulative incidence of relapse（CIR）, and cumulative incidence of acute and chronic graft-versus-host disease（aGVHD and cGVHD）were compared between the groups.

**Results:**

The median follow-up was 33.4 months（95％ *CI*: 31.2–36.1 months）. The 3-year OS rate was significantly lower in older than in younger recipients（75.5％ ± 2.5％ *vs* 83.1％ ± 2.2％, *HR*＝1.59, *P*＝0.007）. The 3-year NRM was higher in older recipients［13.3％ ± 1.9％ *vs* 8.5％ ± 1.6％, univariable subdistribution *HR*（*SHR*）＝1.61, *P*＝0.044］. This association did not reach conventional statistical significance after multivariable adjustment（adjusted *SHR*＝1.59, 95％ *CI*: 0.99–2.54, *P*＝0.053）; however, the stable effect estimate indicated clinical relevance. No statistically significant between-group differences were observed in CIR or in the cumulative incidences of grade Ⅱ–Ⅳ aGVHD and cGVHD. Infection was the leading cause of NRM in both groups, whereas the proportions of deaths attributable to organ dysfunction and bleeding were higher among older recipients.

**Conclusion:**

After propensity score matching, older recipient age remained independently associated with poorer OS. This unfavorable outcome was primarily characterized by an increased risk of NRM, rather than that of relapse.

异基因造血干细胞移植（allo-HSCT）是恶性血液病的有效治疗手段[1]–[2]。随着移植技术的飞速发展，allo-HSCT的安全性及有效性得到了显著提升，适应证范围也随之扩大[3]。国外研究显示，接受allo-HSCT的老年患者比例呈上升趋势[4]。在我国，随着人口老龄化加速、减低强度预处理方案的推广，老年患者接受allo-HSCT的需求也进一步增加[5]。

在临床实践当中，老年患者在确诊时往往具有不同于年轻患者的疾病特征[6]–[8]。受者年龄大多时候和晚期疾病状态、较差的状态评分等不利因素相互交织在一起，使其本身的独立效应变得难以辨识[9]。现有研究大多没能充分地调整上述混杂因素，使受者年龄的效应识别长期受到限制：老年患者的移植结局更差，是因为他们年龄本身存在生物学劣势，如器官功能更差、并发症更多，还是因为他们的疾病特征更差、复发风险更高？是需要去甄别和回答的问题。

我们采用多中心回顾性设计，通过倾向性评分匹配（PSM）平衡年轻受者（≤35岁）与老年受者（≥50岁）在11项关键协变量上的分布，从而评估受者年龄对移植结局的独立影响。本研究旨在回答：受者年龄在调整混杂因素后是否仍为移植的危险因素？若是危险因素，其不良影响主要源于较高的复发率，还是较高的非复发死亡率（NRM）？期望为老年患者的移植风险咨询、个体化治疗决策及未来临床干预研究提供更可靠的临床证据。

## 对象与方法

一、研究设计与人群

本研究为一项回顾性、多中心队列研究。共筛选2018年1月1日至2023年12月31日期间，在中国5个移植中心接受单次allo-HSCT的≤35岁或≥50岁的血液系统恶性肿瘤患者1 619例。疾病类型包括急性髓系白血病（AML）、急性淋巴细胞白血病（ALL）、骨髓增生异常综合征（MDS）。纳入患者具备完整的基线信息、移植特征及关键预后事件数据，排除接受二次或以上allo-HSCT及关键变量［受者年龄、原发疾病诊断、急性或慢性移植物抗宿主病（aGVHD或cGVHD）发生情况］缺失的患者。

本研究以≤35岁为年轻受者组、≥50岁为老年受者组，并排除了中间年龄段（36～49岁）的患者。选择50岁作为老年受者组界值，是因为在我国临床实践中，这通常是需要转向减低强度预处理的“相对高龄”节点；近期两项国际大样本研究也使用了相同的年龄界值[10]–[11]。排除中间年龄段的目的在于最大化两组间的生物学差异，以期在协变量匹配后，能更纯粹地甄别出年龄本身的独立预后价值。

本研究经陆军军医大学第二附属医院医学伦理委员会批准（批件号：2025-研第287-02），获得患者知情同意豁免，已在中国临床试验注册中心注册（注册号：ChiCTR2500108709）。

二、变量定义

1. 主要暴露变量：主要暴露变量为受者年龄，根据研究设计将其二分类为年轻受者组（≤35岁）与老年受者组（≥50岁）。

2. 结局变量：本研究的主要终点为总生存（OS）期，定义为从移植日至任何原因死亡或末次随访的时间。次要终点包括：无事件生存（EFS）期，事件定义为死亡或复发；NRM；累积复发率（CIR）；Ⅱ～Ⅳ度aGVHD累积发生率和cGVHD的累积发生率。

3. 协变量：共11项，包括受者性别（男、女）、疾病类型（AML、ALL、MDS）、移植前疾病状态［完全缓解（CR）、部分缓解（PR）、未缓解（NR）等］、HCT合并症指数（HCT-CI）评分（0分、1～2分、3分及以上、未知）、供者年龄（连续变量）、HLA相合程度（全相合、不全相合）、供者类型（同胞全相合供者、亲缘单倍体供者、无关供者）、供受者血型相合情况（相合、次要不合、主要不合、双向不合、Rh不合）、供受者性别匹配（男供男、男供女、女供女、女供男）、干细胞来源（外周血+骨髓、外周血）、预处理方案强度（标准清髓、降低强度、增强强度、未知）。连续变量无缺失，分类变量中的“未知”作为独立类别纳入分析，以保留样本量。

三、PSM

为均衡两组患者的基线协变量，采用马氏距离最近邻匹配法进行1∶1无放回匹配。倾向评分通过多因素Logistic回归模型计算得出。在该模型中，受者年龄分组为因变量，前述11项协变量为自变量。其中，疾病类型、干细胞来源、HCT-CI评分、预处理强度、供者类型及HLA相合程度实施精确匹配，确保这些因素在两组间完全一致；分类变量（如移植前疾病状态、HCT-CI评分等）中的“未知”作为独立类别纳入模型，以保留样本量。

匹配效果的评估采用标准化均数差（SMD），以SMD<0.1作为组间均衡性良好的判断标准。考虑到临床实际，部分变量SMD略高于0.1仍可接受，并将在后续多因素分析中校正。匹配后，共694例患者（两组各347例）进入最终分析队列。

四、统计学处理

基于匹配后的694例患者进行统计分析。

分类变量用频数（百分比）描述，组间比较用卡方检验或Fisher精确检验；连续变量用均值±标准差描述，组间比较用Mann-Whitney *U*检验。

OS和EFS用Kaplan-Meier法估计，组间比较用Log-rank检验，用Cox比例风险模型计算风险比（*HR*）及其95％置信区间（*CI*）。非复发死亡、复发和GVHD作为竞争风险事件（其中将复发视为非复发死亡的竞争风险，非复发死亡视为复发的竞争风险，死亡视为GVHD的竞争风险），用Fine-Gray竞争风险模型估计累积发生率，组间比较用Gray检验，计算亚分布风险比（*SHR*）及其95％*CI*。

在匹配后队列中，多因素Cox/Fine-Gray模型的校正变量基于临床已知预后因素及匹配后SMD>0.1的变量确定。为处理供者年龄的残余不平衡，在针对OS和NRM的多因素模型中加入“受者年龄组×供者年龄”的交互项，并按供者年龄中位数进行分层分析；为检验中心间异质性的潜在影响，我们构建了以中心作为随机效应（frailty term）的混合效应Cox模型来分析OS。此外，针对移植前疾病状态、HCT‑CI评分及预处理强度中“未知”类别可能引入的偏倚，本研究将上述三个变量的“未知”类别作为整体，通过最佳-最差病例敏感性分析同时进行极端赋值（最差场景下年轻受者组的“未知”统一赋值为最佳状态、老年受者组赋值为最差状态，最佳场景则反向操作），从而构造两种综合偏倚场景以最大化检验结论稳健性。所有检验均为双侧，*P*<0.05为差异有统计学意义。使用R软件（版本4.5.2）进行分析及绘图。

## 结果

一、患者基线特征与匹配效果

PSM匹配后共纳入694例患者，年轻受者和老年受者各347例。除供者年龄外（SMD＝0.289），其余纳入匹配的主要协变量达到较好平衡；在后续多因素分析中已予以调整（表1）。

**表1:** 倾向性评分匹配后年轻与老年异基因造血干细胞移植受者的基线特征比较

基线特征	总队列 （694例）	年轻受者 （347例）	老年受者 （347例）	*SMD*	*P*值
受者性别［例（％）］				0.041	0.647
男性	387（55.8）	190（54.8）	197（56.8）		
女性	307（44.2）	157（45.2）	150（43.2）		
疾病类型［例（％）］				<0.001	1.000
急性髓系白血病	466（67.1）	233（67.1）	233（67.1）		
急性淋巴细胞白血病	120（17.3）	60（17.3）	60（17.3）		
骨髓增生异常综合征	108（15.6）	54（15.6）	54（15.6）		
移植前疾病状态［例（％）］				0.209	0.125
完全缓解	514（74.1）	253（72.9）	261（75.2）		
部分缓解	12（1.7）	7（2.0）	5（1.4）		
未缓解	1（0.1）	1（0.3）	0（0）		
疾病稳定	2（0.3）	1（0.3）	1（0.3）		
复发/难治	38（5.5）	13（3.7）	25（7.2）		
未知	127（18.3）	72（20.7）	55（15.9）		
HCT-CI评分［例（％）］				<0.001	1.000
0分	316（45.5）	158（45.5）	158（45.5）		
1～2分	248（35.7）	124（35.7）	124（35.7）		
3分及以上	56（8.1）	28（8.1）	28（8.1）		
未知	74（10.7）	37（10.7）	37（10.7）		
供者类型［例（％）］				<0.001	1.000
同胞全相合供者	212（30.5）	106（30.5）	106（30.5）		
单倍体相合供者	450（64.8）	225（64.8）	225（64.8）		
无关供者	32（4.6）	16（4.6）	16（4.6）		
HLA相合程度［例（％）］				<0.001	1.000
完全相合	242（34.9）	121（34.9）	121（34.9）		
不完全相合	452（65.1）	226（65.1）	226（65.1）		
受者-供者血型组合［例（％）］				0.174	0.239
相合	427（61.5）	226（65.1）	201（57.9）		
次要不合	123（17.7）	54（15.6）	69（19.9）		
主要不合	112（16.1）	54（15.6）	58（16.7）		
双向不合	31（4.5）	13（3.7）	18（5.2）		
Rh不合	1（0.1）	0（0）	1（0.3）		
受者-供者性别组合［例（％）］				0.182	0.127
男-男	264（38.0）	133（38.3）	131（37.8）		
男-女	191（27.5）	107（30.8）	84（24.2）		
女-女	112（16.1）	48（13.8）	64（18.4）		
女-男	127（18.3）	59（17.0）	68（19.6）		
预处理强度［例（％）］				<0.001	1.000
标准	544（78.4）	272（78.4）	272（78.4）		
减低强度	56（8.1）	28（8.1）	28（8.1）		
增强强度	90（13.0）	45（13.0）	45（13.0）		
未知	4（0.6）	2（0.6）	2（0.6）		
移植物来源［例（％）］				<0.001	1.000
外周血+骨髓	272（39.2）	136（39.2）	136（39.2）		
外周血	422（60.8）	211（60.8）	211（60.8）		
供者年龄（岁，*x*±*s*）	34.73±12.62	32.94±11.81	36.57±13.29	0.289	<0.001

**注** HCT-CI：HCT合并症指数；年轻受者：受者年龄≤35岁；老年受者：受者年龄≥50岁

二、生存结局

中位随访时间为33.4（95％ *CI*：31.2～36.1）个月。老年受者组的生存结局显著更差，其3年OS率为（75.5±2.5）％，低于年轻受者组的（83.1±2.2）％。Kaplan-Meier分析显示，老年受者与更差的OS显著相关（*HR*＝1.59，95％ *CI*：1.13～2.24，*P*＝0.007），其EFS呈现劣于年轻受者的趋势（*HR*＝1.30，95％ *CI*：0.99～1.72，*P*＝0.059）（图1）。

**图1:**
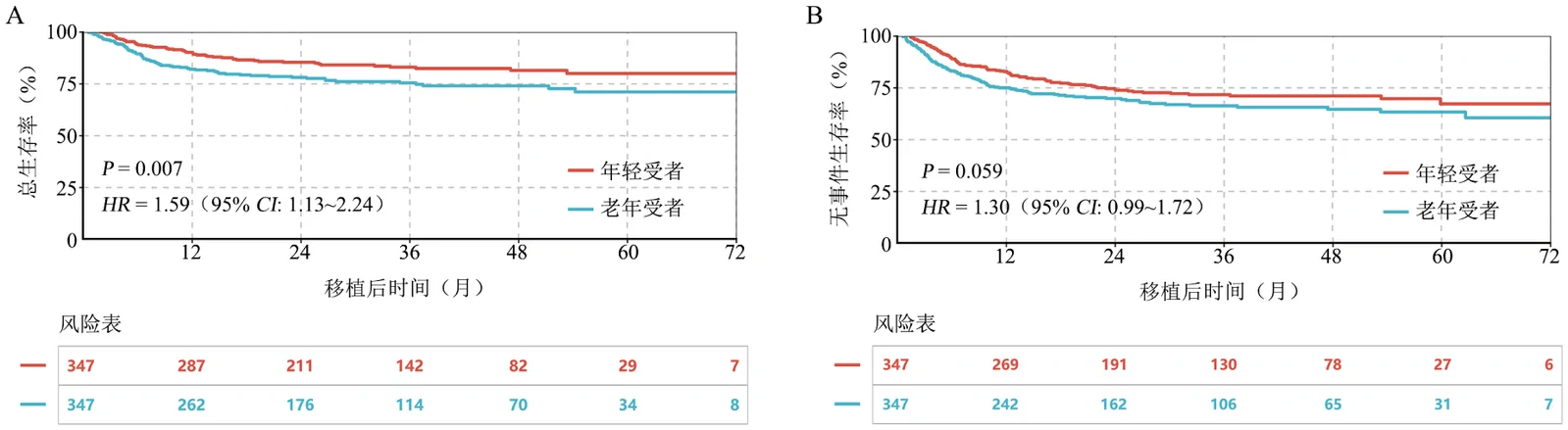
年轻与老年受者异基因造血干细胞移植后总生存（A）与无事件生存（B）的Kaplan-Meier曲线比较 **注** 年轻受者：受者年龄≤35岁；老年受者：受者年龄≥50岁

三、非复发死亡与复发

老年受者组的3年累积NRM为（13.3±1.9）％，显著高于年轻受者组的（8.5±1.6）％（*SHR*＝1.61，95％ *CI*：1.01～2.57，*P*＝0.044）。相比之下，两组的3年CIR相当［（20.4±2.3）％对（19.8±2.3）％，*SHR*＝1.08，95％*CI*：0.77～1.53，*P*＝0.640］（图2）。提示老年受者的生存劣势并不是由于复发风险高导致的。

**图2:**
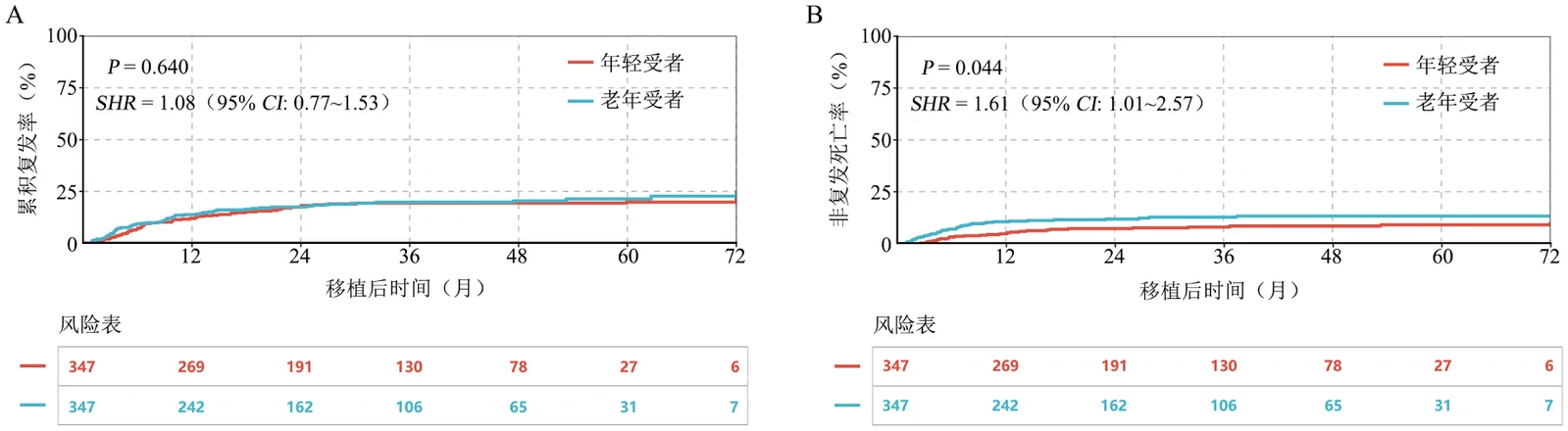
年轻与老年受者异基因造血干细胞移植后CIR（A）与NRM（B）的竞争风险曲线比较 **注** 年轻受者：受者年龄≤35岁；老年受者：受者年龄≥50岁；CIR：累积复发率；NRM：非复发死亡率

在匹配后的694例患者中，年轻受者组共发生29例非复发死亡（占该组8.4％），老年受者组共发生44例非复发死亡（占该组12.7％），两组非复发死亡的差异未达统计学显著性（*P*＝0.083）。为进一步探究老年受者NRM升高的潜在原因，对其具体死因构成进行归类分析。

如表2所示，感染是两组最主要的非复发死亡原因，分别占年轻受者组和老年受者组非复发死亡的48.3％（14/29）和38.6％（17/44）（*P*＝0.473）。老年受者组因器官功能障碍导致的死亡比例（15.9％，7/44）高于年轻受者组（10.3％，3/29），但差异无统计学意义（*P*＝0.730）。值得注意的是，出血相关死亡仅见于老年受者（6.8％，3/44），而年轻受者无一例发生；但由于例数极少，组间比较差异无统计学意义（*P*＝0.272）。两组在GVHD相关死亡（*P*＝0.524）及死因不明（*P*＝0.781）方面的构成比差异亦无统计学意义。

**表2:** 倾向性评分匹配后年轻与老年异基因造血干细胞移植受者非复发死亡原因构成［例（％）］

死因	年轻受者组 （347例）	老年受者组 （347例）	*P*值
非复发死亡	29（8.4）	44（12.7）	0.083
感染	14（48.3）	17（38.6）	0.473
器官功能障碍	3（10.3）	7（15.9）	0.730
GVHD相关	6（20.7）	6（13.6）	0.524
出血	0（0）	3（6.8）	0.272
未知/其他	6（20.7）	11（25.0）	0.781

**注** 总体非复发死亡以例数（占受者年龄分组的百分比）表示，具体死因以例数（占该组非复发死亡总数的百分比）表示。*P*值基于Fisher精确检验，比较两组在各死因类别中的构成比差异。器官功能障碍包括肝静脉闭塞病、肾衰竭、心力衰竭、呼吸衰竭及多器官功能衰竭；出血包括颅内出血及消化道出血。因各组非复发死亡事件数有限，死因构成比的统计学检验效能较低，结果需谨慎解读

总体来看，老年受者组的非复发死亡负担更重，但两组的死因基本一致——感染、器官功能障碍、出血，且在老年受者组中的实际死亡例数都多于年轻受者组。提示老年受者NRM升高的原因，可能不是某种并发症的单一作用，而是对移植相关并发症的整体耐受性下降。

四、GVHD

两组间Ⅱ～Ⅳ度aGVHD和cGVHD的发生率差异无统计学意义。年轻受者和老年受者100 d Ⅱ～Ⅳ度aGVHD累积发生率分别为（24.5±2.3）％和（21.6±2.2）％（*SHR*＝0.87，95％ *CI*：0.64～1.17，*P*＝0.350）。两组的3年cGVHD累积发生率分别为（45.0±2.8）％和（43.4±2.8）％（*SHR*＝0.90，95％ *CI*: 0.71～1.13，*P*＝0.360）（图3）。

**图3:**
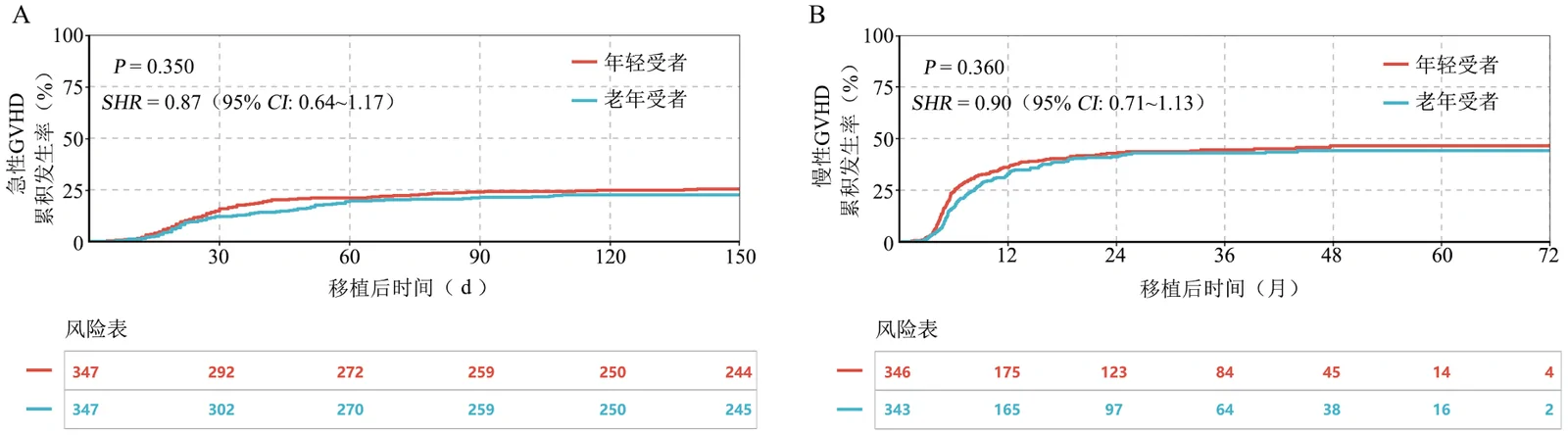
年轻与老年受者异基因造血干细胞移植后Ⅱ～Ⅳ度急性GVHD（A）与慢性GVHD（B）累积发生率的竞争风险曲线比较（死亡被视为GVHD的竞争风险） **注** 年轻受者：受者年龄≤35岁；老年受者：受者年龄≥50岁；GVHD：移植物抗宿主病

五、单因素及多因素分析

在单因素分析中，老年受者与OS较差（*HR*＝1.59，95％*CI*：1.13～2.24，*P*＝0.007）、NRM较高（*SHR*＝1.61，95％*CI*：1.01～2.57，*P*＝0.044）显著相关，其EFS亦呈现劣于年轻受者的趋势（*HR*＝1.30，95％*CI*：0.99～1.72，*P*＝0.059），而CIR、cGVHD、aGVHD的组间差异均无统计学意义（均*P*>0.05）（表3）。

**表3:** 受者年龄分组对移植结局的单因素及多因素分析结果

研究终点	单因素分析		多因素分析	
	*HR*/*SHR*（95％*CI*）	*P*值	*aHR*/*aSHR*（95％*CI*）	*P*值
总生存^a^	1.59（1.13～2.24）	0.007	1.55（1.10～2.18）	0.013
无事件生存^a^	1.30（0.99～1.72）	0.059	1.27（0.97～1.68）	0.087
非复发死亡^b^	1.61（1.01～2.57）	0.044	1.59（0.99～2.54）	0.053
累积复发^b^	1.08（0.77～1.53）	0.640	1.03（0.72～1.47）	0.880
慢性GVHD^b^	0.90（0.71～1.13）	0.360	0.91（0.72～1.15）	0.440
Ⅱ～Ⅳ度急性GVHD^b^	0.87（0.64～1.17）	0.350	0.87（0.64～1.17）	0.360

**注** GVHD：移植物抗宿主病；参考年龄组为年轻受者组，^a^为Cox比例风险模型，^b^为Fine-Gray竞争风险模型

进一步对单因素分析中*P*<0.1或临床意义重要的协变量（包括受者性别、供受者性别关系、疾病状态、预处理强度、HLA相合情况、血型相容性、供者类型及HCT-CI评分等）进行校正后，多因素分析结果显示：老年受者仍为OS不良的独立危险因素（*aHR*＝1.55，95％*CI*：1.10～2.18，*P*＝0.013）；其NRM风险亦呈升高趋势，但在多因素校正后未达到传统统计学显著性阈值（*aSHR*＝1.59，95％ *CI*：0.99～2.54，*P*＝0.053），效应量稳定提示临床意义；而EFS的组间差异未达到传统统计学意义水平（*aHR*＝1.27，95％*CI*：0.97～1.68，*P*＝0.087）。在本研究中，未观察到老年受者对CIR、cGVHD及aGVHD的独立影响（均*P*>0.05）（表3）。多因素模型完整分析结果见附表1（**扫描本文首页二维码查阅**）。

六、供者年龄交互作用与中心异质性检验

匹配后供者年龄在两组间仍有轻度不平衡（SMD＝0.289）。为评估其对主要结论的影响，我们在OS和NRM的多因素模型中分别加入“受者年龄组×供者年龄”的交互项。结果显示，交互项对OS（交互*P*＝0.954）和NRM（交互*P*＝0.600）均无统计学意义。进一步按供者年龄中位数将全队列分为年轻供者亚组（≤35岁，388例）与年长供者亚组（>35岁，306例），发现老年受者在两个亚组中的OS和NRM风险一致升高：年轻供者亚组中，OS *HR*＝1.60（95％*CI*: 1.01～2.53），NRM *SHR*＝1.63（95％ *CI*: 0.84～3.13）；年长供者亚组中，OS *HR*＝1.58（95％ *CI*: 0.94～2.66），NRM *SHR*＝1.58（95％ *CI*: 0.82～3.05）。各亚组效应方向一致，效应量稳定（附表2）（**扫描本文首页二维码查阅**）。

为评估各移植中心间的异质性，我们以中心为随机效应拟合了混合效应Cox模型。结果显示，中心间差异未达统计学显著性（frailty项*P*＝0.057），校正中心效应后老年受者组OS的*HR*为1.59（95％ *CI*: 1.13～2.24，*P*＝0.008），与主分析完全一致，提示各中心间结局无显著异质性，结论稳健。

七、敏感性分析

为检验协变量中存在的缺失数据（标记为“未知”）对研究结论稳健性的潜在影响，我们针对OS、NRM和CIR终点进行了最佳-最差场景敏感性分析（表4）。在两种极端赋值假设下，多因素分析显示老年受者组OS和NRM的效应估计方向均总体指向较高风险，CIR亦始终未见明确的组间差异。OS在最差场景下统计学显著性减弱，但效应方向未发生逆转；NRM的结果在各场景下均未达到统计学显著性；CIR结论保持一致。总体而言，缺失数据的不同极端处理未改变研究的主要结论，提示结果具有一定稳健性。

**表4:** 年轻与老年异基因造血干细胞移植受者主要终点的最佳-最差场景敏感性分析

结局	*aHR*/*aSHR*（95％ *CI*）	*P*值
总生存		
原始	1.55（1.10～2.18）	0.013
最差场景	1.36（0.95～1.95）	0.095
最佳场景	1.55（1.08～2.22）	0.017
非复发死亡		
原始	1.59（0.99～2.54）	0.053
最差场景	1.60（0.99～2.59）	0.055
最佳场景	1.47（0.87～2.48）	0.150
累积复发		
原始	1.03（0.72～1.47）	0.880
最差场景	0.90（0.60～1.33）	0.590
最佳场景	1.36（0.92～2.03）	0.130

**注** 最差场景：年轻受者组“未知”赋值为最佳临床状态，老年受者组“未知”赋值为最差状态；最佳场景反向赋值

## 讨论

这项多中心回顾性研究利用PSM均衡了两组患者的基线特征，以尽可能降低选择偏倚，并评估受者年龄对allo-HSCT结局的影响[12]。结果显示，高龄（≥50岁）是OS的独立危险因素，老年受者的EFS也呈现出较差的趋势。本研究中老年受者的生存劣势主要由NRM增高驱动：尽管多因素分析中NRM未达到传统统计学显著性阈值（*P*＝0.053），但其效应量与单因素分析结果高度一致（*aSHR*与*SHR*接近），提示老年受者NRM风险升高的趋势较为稳定，值得在更大样本中进一步验证。此外，两组在CIR、aGVHD、cGVHD方面均未见显著差异，说明在均衡主要基线特征后，老龄本身并未增加主要移植并发症的风险。

本研究基于中国多中心数据评估了受者年龄在allo‑HSCT中的独立效应。在以单倍体移植为主的中国移植体系下，受者年龄的预后价值仍缺乏经混杂因素均衡后的大样本证据[13]–[15]。通过排除36～49岁中间年龄段，本研究将年轻受者组与老年受者组的生物学年龄差距尽可能拉开，从而更清楚地分离出年龄本身对预后的影响。本研究中，老年受者的生存劣势主要源于NRM增高而非复发，这一趋势为老年移植患者的临床管理提供了一定的参考——即在强化抗白血病效应（GVL）的同时，移植耐受性的改善可能同样不应被忽视。

导致老年患者NRM升高的原因可能是多方面的。器官储备功能下降是老年患者不良结局的核心。尽管HCT-CI评分在匹配后分布均衡，但相同分值的老年患者实际器官功能往往更差[16]，而本研究中老年受者组器官功能障碍和出血相关死亡的占比在数值上也高于年轻受者组。这一方向性的差异提示器官储备在非复发死亡中的潜在作用，但受限于死因构成的例数有限，这一推断仍需更大样本量加以验证。其次，免疫衰老导致的T细胞重建延迟、免疫监视功能减弱，使得老年受者感染易感性显著增加[17]–[18]。值得注意的是，感染虽为两组非复发死亡的首要原因，但老年受者组感染的绝对例数更多、构成比却较低，这与器官功能障碍及出血等非感染性死亡占比的上升有关。提示老年受者NRM的增高可能是多种并发症共同作用的结果，而非某一类事件的单独效应，其具体构成仍需更大样本量加以验证。此外，年龄相关的药代动力学变化，如白消安等预处理药物在老年患者体内清除率降低、毒性暴露延长[19]，也可能加重器官损伤。本研究未观察到受者年龄与GVHD发生率存在显著关联，考虑到两组患者在基线特征上具有可比性，受者年龄对GVHD风险的独立影响在本研究条件下可能未充分体现，其潜在效应仍有待进一步验证。

西班牙GETH-TC注册研究报道了529例≥65岁allo-HSCT受者的结局，4年OS率为43.4％，NRM达32.0％[13]。与之相比，本队列老年受者组3年OS率为（75.5±2.5）％，NRM为（13.3±1.9）％。两组数据的差异可能与年龄界值（≥50岁对≥65岁）、预处理策略及支持治疗强度、以及供者来源构成（本组单倍体移植占64.8％）不同有关。值得关注的是，GETH-TC研究中ECOG评分、HCT-CI和疾病风险指数（DRI）对生存的影响超过了实际年龄本身[13]，这提示：年龄对预后的效应可能正是通过器官储备和合并症负担间接实现的。上述对比也提示，我国当前的老年allo-HSCT实践体系可能具有一些独特的预后优势。

本研究的发现对临床决策具有一定参考价值。老年患者即使在良好的疾病状态下接受了年轻供者的移植，年龄本身仍是预后不良的独立因素。在本队列中，老年受者器官功能障碍及出血相关死亡的例数多于年轻受者，这一趋势提示，预处理阶段对肝肾功能及凝血状态的评估和个体化调整可能有助于降低此类事件的风险。考虑到感染仍是两组最主要的非复发死亡原因，移植后对肾、肝及凝血指标的动态监测，以及抗感染方案的个体化调整，同样不可忽视。未来研究可在本研究的基础上，开展年龄分层的前瞻性临床试验，并尝试将老年综合评估指标与生物标志物整合，构建适用于中国老年allo‑HSCT受者的预后模型。

当然，本研究也存在一些局限。首先，尽管PSM已较好地平衡了两组基线，但一些无法测量的混杂因素（如衰弱程度、营养状况）可能仍有残余影响；部分变量存在“未知”类别，虽然敏感性分析显示极端赋值下OS和NRM的效应方向保持一致，CIR在所有场景下均未达统计学显著性，但偏倚不能完全排除。其次，匹配后供者年龄在两组间仍有轻度不平衡（SMD＝0.289），我们通过交互检验和亚组分析证实了该不平衡未扭曲主要结论，不过这一残余差异提示PSM在处理连续型关键变量时仍有优化空间。中心间的异质性也在混合效应模型中得到了检验，frailty项未达统计学意义（*P*＝0.057），校正后年龄效应量稳定。另一个需要注意的问题是，NRM在多因素分析中未达到传统的显著性水平（*P*＝0.053），尽管效应量与单因素分析高度一致，提示这一关联可能因事件数有限而未能达到统计学阈值；受限于相同的原因，老年亚组内NRM高危因素的探索性分析也未能发现独立预测因子（附表3）（**扫描本文首页二维码查阅**）。

此外，本研究在人群代表性和时效性方面亦有不足。因设计需要排除36～49岁中间年龄段，结论向更广泛的年龄谱系推广时需谨慎；数据来源于国内5个移植中心，人群代表性较好但结果的跨中心、跨人群适用性仍需验证。本研究的中位随访时间为33.4（95％*CI*：31.2～36.1）个月，但cGVHD具有延迟出现和病程迁延的特点，部分患者在移植后2～3年仍可新发或转为广泛型cGVHD，因此cGVHD的3年累积发生率可能存在一定程度的低估。

本研究中，受者高龄是allo-HSCT后OS不良的危险因素，老年受者较差的生存结局可能与NRM增高有关，而未观察到与复发风险的明确关联。这一发现提示，改善老年患者预后的关键可能在于提升移植耐受性，而不仅仅是强化GVL。在临床实践中，围绕耐受性评估、感染防控和器官功能保护来优化移植策略，或许比单纯追求更强烈的GVL效应更为现实。未来，将老年综合评估纳入移植前决策并在此基础上制定个体化移植方案，可能有助于进一步改善这一持续增长的老年患者群体的预后。

## Supplementary Material


